# Contemporary Drought and Future Effects of Climate Change on the Endangered Blunt-Nosed Leopard Lizard, *Gambelia sila*

**DOI:** 10.1371/journal.pone.0154838

**Published:** 2016-05-02

**Authors:** Michael F. Westphal, Joseph A. E. Stewart, Erin N. Tennant, H. Scott Butterfield, Barry Sinervo

**Affiliations:** 1 Hollister Field Office, U.S. Bureau of Land Management, Hollister, California, United States of America; 2 Department of Ecology and Evolutionary Biology, University of California at Santa Cruz, Santa Cruz, California, United States of America; 3 Lands Unit, Central Region, California Department of Fish and Wildlife, Fresno, California, United States of America; 4 The Nature Conservancy, San Francisco, California, United States of America; Smithsonian Conservation Biology Institute, UNITED STATES

## Abstract

Extreme weather events can provide unique opportunities for testing models that predict the effect of climate change. Droughts of increasing severity have been predicted under numerous models, thus contemporary droughts may allow us to test these models prior to the onset of the more extreme effects predicted with a changing climate. In the third year of an ongoing severe drought, surveys failed to detect neonate endangered blunt-nosed leopard lizards in a subset of previously surveyed populations where we expected to see them. By conducting surveys at a large number of sites across the range of the species over a short time span, we were able to establish a strong positive correlation between winter precipitation and the presence of neonate leopard lizards over geographic space. Our results are consistent with those of numerous longitudinal studies and are in accordance with predictive climate change models. We suggest that scientists can take immediate advantage of droughts while they are still in progress to test patterns of occurrence in other drought-sensitive species and thus provide for more robust models of climate change effects on biodiversity.

## Introduction

The urgent need to predict the effects of climate change demands innovative approaches and the flexibility to exploit contemporary patterns of variation. A drought of historic severity in Western North America [1] afforded a rare glimpse into a future where droughts are more common and more extreme. Severe drought has been proposed as a major component of near-term future climate change in the North American Southwest [2–3]. Historical reconstruction of past droughts has been an important strategy for estimating impacts of droughts to come [3, 4–5]. Few studies, however, have investigated contemporary droughts in real time with respect to future climate change [6–7]. We here present results of a study in which we took advantage of a drought in-process to test models of the effect of climate change on the endangered blunt-nosed leopard lizard (*Gambelia sila*) across the species' range. We provide this report in hopes of inspiring other scientists worldwide to exploit the immediate potential of droughts still in progress to provide insights that may help optimize models of climate change effects on priority resources.

An accelerated rate of species extinction, particularly of already-vulnerable species [8], has been a proposed effect of climate change [9]. We therefore need models that will help us accurately predict patterns of species persistence and extinction and determine appropriate climate refugia [10–14]. The integration of theoretical and empirical approaches can be an effective strategy for predicting species persistence. For example, richness regression models have been combined with species occurrence point data [15], while the performance of correlative approaches have been compared to mechanistic approaches for predicting species distribution [16]. Lizard thermal requirements have been effectively linked with patterns of observed extinction at global scales during the historical period of climate change (1975–2009) [17]. Some workers have called attention to the necessity of validating climate envelope models with empirical data on species presence to remove uncertainty [18]. Here, we present the results of a short-term study on variation in juvenile recruitment (a surrogate for species persistence) in *G*. *sila* during a historically extreme drought in western North America, which we link both to drought indices and to an ecosystem measure of plant greenness (ie productivity) referred to as the Normalized Difference Vegetation Index (NDVI).

*Gambelia sila* is a Federal and State listed endangered desert species endemic to the San Joaquin Desert, an ecosystem that persists in relict fragments at the periphery of its historical extent [19]. Because xeric environments may be particularly vulnerable to climate change [5], species such as *G*. *sila* that are limited to fragmented desert habitats [20] may be at extreme risk and are good models for projecting climate change effects on desert ecosystems. In 2012, we began developing a spatial model of climate-mediated extinction risk of *G*. *sila* across its historic range. At the time, the drought of 2012–14, the most severe multi-year drought in southwestern North America recorded in the past 1200 years (−14.55 cumulative Palmer Drought Severity Index (PDSI)), was in its third year [1]. The effect of drought on reptilian recruitment dynamics has been well documented [21–28] including in *G*.*sila* [29] and other federally-listed California reptiles [30–31]. All of these studies were the product of intensive multi-year projects at one or two sites. Our aim was to test, in a single season, a model that predicts *G*. *sila* recruitment as a function of winter precipitation [32–33]. To conduct this test we organized a range-wide neonate survey of all known, extant populations of *G*. *sila*. Our overall aims were to investigate the relationship between precipitation-mediated primary productivity and neonate presence among many sites along a precipitation gradient, and to test how closely the spatial pattern of neonate presence fit a model of future climate change predicting increased drought across the range of *G*. *sila*.

## Methods

### Site selection

We selected sites along a latitudinal gradient spanning the range of *G*. *sila*. Because the sites we surveyed represented a majority of the remaining sites known to be currently occupied by *G*. *sila*, random selection of sites was not feasible. All sites fulfilled the following criteria: adults had been positively identified in the survey area in last three years (= “prior occupancy survey”) (S1 Table); no sites had experienced conversion or disturbance in the recent past (<20 yrs); and all sites were located on patches of habitat of sufficient size to have promoted long-term persistence of the population (i.e. >500 ha) [20]. Many of the sites had been the subject of multi-year ecological and genetic studies, confirming the baseline presence of the species.

### Survey Method

We employed a simple presence/absence survey designed to maximize our resources in the narrow temporal window available and to coincide with the period when neonates emerge and begin feeding. We surveyed the sites in optimal thermal conditions (24–38 C) until at least one neonate was observed or until three separate surveys had been completed. Past studies have suggested that 3 survey days are the minimum required to detect *G*. *sila* [34] and neonates tend to be more abundant than adults [29]. The survey methodology at each site was chosen to replicate the method that had produced positive results (i.e. observations of multiple individual lizards) in the prior occupancy survey (S1 Table). Except for one site, both the prior occupancy surveys and the neonate surveys were conducted by the same observers. In most cases both the prior occupancy surveys and neonate surveys consisted of timed driving surveys along established transects, walking surveys using the same methodology, or both. Driving surveys were conducted by slowly (<10km/hour) driving along the roadside with frequent pauses to examine individual animals that were potentially *G*. *sila*. In most of the surveys, lizards were either captured or recorded with a high-resolution camera. Neonate status was inferred by measured snout-to-vent lengths of under 80mm, a consistent measure of age in the fall [34].

### Statistical Analysis

We used logistic regressions, AICc model selection, and t-tests to test the ability of winter precipitation, NDVI (i.e. vegetation biomass), soil texture, and soil pH to predict neonate recruitment. Winter precipitation was extracted from PRISM interpolated weather surfaces (4-km resolution) for the period October 2013 to April 2014 [35]. NDVI, for the same period, was extracted from 250-m resolution MODIS surfaces, averaged across all cells within a 500-m radius. Soil texture (percent sand, silt, and clay) and soil pH were extracted from SSURGO soil maps and averaged across soil horizons. To avoid overfitting, all candidate predictor variables were evaluated in simple logistic regressions. Because our selection of sites was nonrandom, we tested for the potential influence of spatial autocorrelation using Moran's test on the residuals of all regression models [36]. All statistical tests were done in [R] ver. 3.1.2 [37].

This study complies with the University of California, Santa Cruz policy regulating research on animals through a permit to conduct a UCSC Live Vertebrate Animal Study—Non-Biomedical, granted by the UCSC Institutional Animal Use and Care Committee, permit #Sineb1402. Capture of live lizards was authorized by the United States Fish and Wildlife Service via permits TE166383-4 (MFW) and TE5257B-0 (JAES), a Memorandum of Understanding issued to the California Department of Fish and Wildlife (ENT), and by the California Department of Fish and Wildlife via Scientific Collecting Permit SC-2925 (MFW). We ameliorated any suffering of captured animals by allowing only trained personnel to handle them, and by limiting handling and housing time to the minimum necessary to record relevant measurements, then releasing animals immediately at point of capture.

## Results

**Fig 1:**
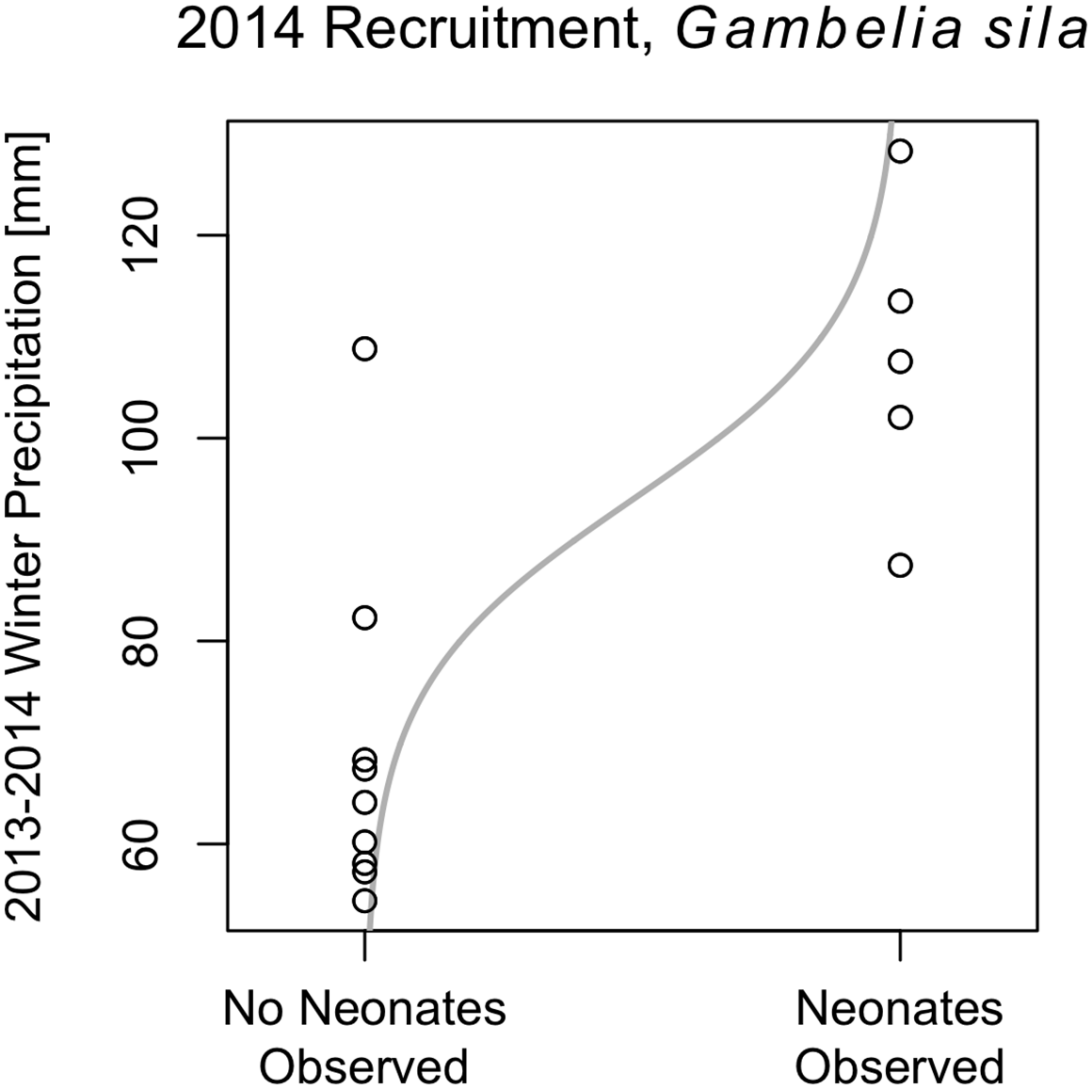
Sites where neonate *G*. *sila* were and were not observed plotted against winter precipitation. The line is predicted probability of observation as derived from a logistic regression fit to the data.

**Fig 2:**
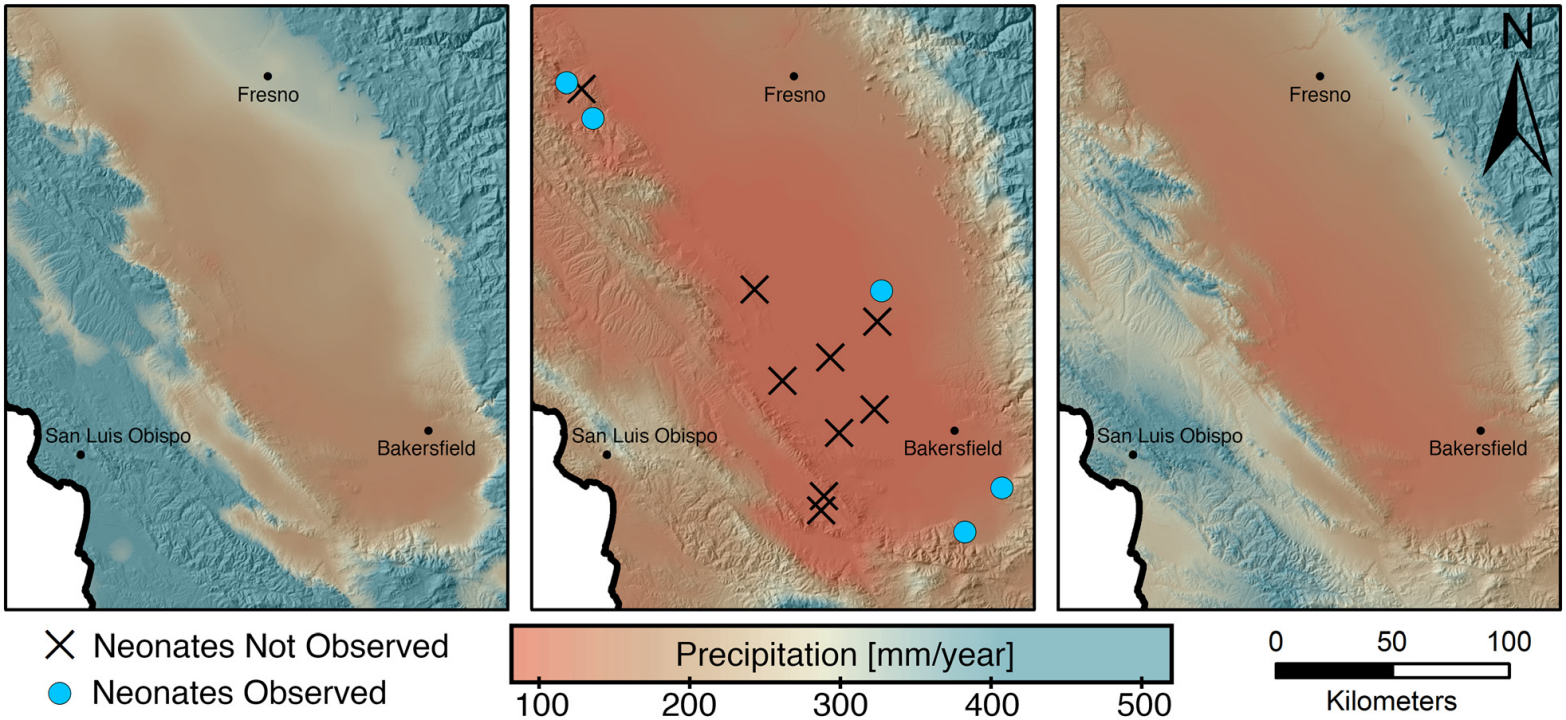
Comparison of past, present and predicted drought patterns and *G*. *sila* recruitment. Map backgrounds are colored by mean annual precipitation for recent (1981–2010, left panel), 2014 water year (middle panel), and projected future (2070–2099, MIROC-ESM RCP8.5, right panel) periods. Results of 2014 surveys for *G*. *sila* neonates are shown in the middle panel.

**Fig 3:**
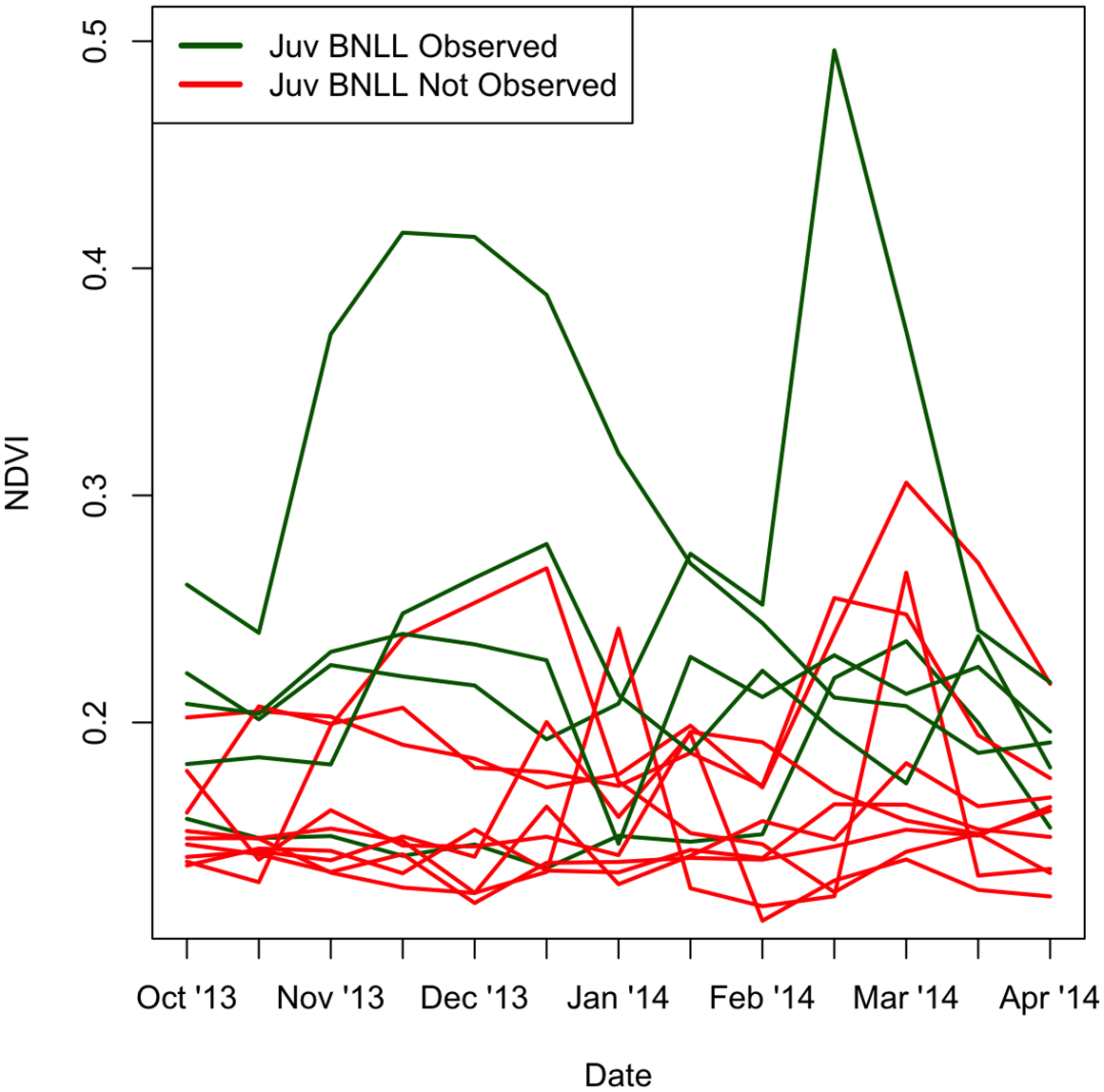
Time series of NDVI values at 14 sites included in this study (16-day temporal resolution). NDVI was extracted from 250-m resolution MODIS grid cells using a 500-m neighborhood around the centroid of BNLL neonate surveys.

We surveyed 14 sites between August 7 and September 24 2014 and detected neonates within the first three days of surveys at seven of them. Winter precipitation over the study sites ranged from 54 mm to 128 mm in 2014. Winter precipitation (Figs 1 and 2) and cumulative winter NDVI (Fig 3) were higher at sites where juvenile *G*. *sila* were observed compared with sites where they were not observed (two-sided t-test, *p* < 0.005 and *p* < 0.05, respectively). All five other candidate variables did not significantly differentiate sites with and without recruitment (two-sided t-test, p > 0.1). Winter precipitation strongly outperformed all other logistic models of recruitment (ΔAICc > 2, S2 Table). Logistic regression of neonate occupancy against winter precipitation (Fig 1), cumulative NDVI (Fig 3), and maximum NDVI revealed strong to significant relationships (ANOVA, *p* < 0.005, *p* < 0.005, p < 0.05, respectively). Soil texture and pH did not statistically differentiate sites where juveniles were observed from sites where they were not observed in logistic regression (ANOVA p > 0.1). Moran’s test failed to detect unaccounted for spatial autocorrelation in the residuals of any of the three best performing models, which measured the effect of precipitation and vegetation (NDVI) on recruitment (|I| < 0.25, p > 0.2). The four worst performing models, which included soil texture and pH, all had unaccounted-for spatial autocorrelation in their residuals (I > 0.25, p < 0.05).

## Discussion and Conclusions

By conducting rapid surveys in a systematic manner we were able to exploit a contemporary weather event to test a model of the effect of climate change on persistence of an endangered lizard in a single season. The absence of neonates from multiple sites provides evidence that reproduction and recruitment at these sites was low or failed altogether in 2014. The correlation of neonate presence with both winter precipitation and NDVI suggests that drought may have played a role in reproductive failure of *G*. *sila* at those sites. Given the predicted increase in drought events due to climate change across the range of *G*. *sila*, we can cautiously interpret our results to infer that climate change poses a credible risk to the persistence of the species over a significant portion of its range. However, because our study is correlative in design, our inference is necessarily limited, and unknown confounding elements could have affected our outcome. Random sampling across a larger set of populations, had such been possible, would have reduced potential unexamined biases arising from site selection. We do believe, however, that our results validate our spatial approach to climate change model testing. Moreover, our results demonstrate a strong effect of winter precipitation in all known population segments of the current species distribution and as such are a thorough assessment across the remaining range.

More work needs to be done to identify the mechanisms that mediate the correlation between winter precipitation and neonate recruitment. Reduced soil moisture, increased soil temperatures, and other effects correlated with winter precipitation and NDVI could have affected egg-laying and egg survival [38–39]. Food availability could also play a role. A potential link between lizard fecundity and the availability of grasshoppers (their primary food source) has been suggested [32–33]. A positive correlation between grasshopper abundance and primary productivity [40] provides further support for the potential interaction between precipitation and leopard lizard recruitment. Directly measuring grasshopper abundance in the field would bolster the causal link between drought and lizard reproduction and should be conducted in the future.

The relationship between habitat fragmentation and effect of climate change will be of crucial importance in predicting the future persistence of *G*. *sila* [20]. Larger patches will likely sustain larger populations which in turn promote intrapopulation genetic diversity, increasing the probability that at least some individuals will reproduce during even a severe drought. Within-patch heterogeneity in elevation and latitude may be even more crucial, as lizards from regions less affected by droughts would be able to recolonize regions where recruitment had failed in a given season. If severe enough (and exacerbated by anthropogenic effects like water table draw-down for agriculture or habitat fragmentation), drought can ultimately generate the extinction of local populations, which may never be recolonized if the population segment is disjunct from other potential source populations [30]. One key benefit of our study is the identification of potential climate change refugia for *G*. *sila* in the Panoche Valley region and Carrizo Plain National Monument in the coast ranges, the Tejon Ranch region in the southern Sierra Nevada foothills and northern Transverse Ranges, and the eastern San Joaquin Valley (e.g. Pixley National Wildlife Refuge) (Fig 2). Linking these refugia with less drought resilient patches on the San Joaquin Valley floor will likely be crucial to the long-term persistence of the species.

Robustly predicting the persistence of *G*. *sila* in the face of climate change will depend on the accurate measurement of multiple interacting habitat features, and would be best accomplished by the construction of an integrated, spatially explicit habitat suitability model that takes into account patch size as well as other crucial habitat elements, including soil, vegetation, land use history, grazing, habitat patch size, and risk of habitat conversion. The present study was a component of a larger effort on the part of the authors to construct just such a model, and contributes a crucial element to the larger model, namely, discovering a minimum rainfall / NDVI threshold for reproductive success in *G*. *sila*.

Although research focusing narrowly on the proximate causes of recruitment failure in *G*. *sila* is a high priority, the results of our study can also gain depth from repeated surveys of *G*. *sila* presence over the remainder of the drought and especially in the years following the cessation of the drought, when we would expect to see a positive response to rainfall in surviving populations. Broadening the study to include other ectotherms can make our conclusions more general. Indeed, synthesizing the results of multiple surveys targeting any drought-sensitive species in the affected areas would contribute to a deeper understanding and perhaps more accurate picture of the effects of climate change. We are currently conducting studies in the San Joaquin Desert on multiple species, including amphibians, endemic desert snakes, shrubs, and small mammals. We urge other scientists to bring resources to bear in future droughts in order to better understand future impacts of climate change on biodiversity.

## Supporting Information

S1 Table Table of surveyed localities and winter precipitation. Binary data in column titled “Neonates observed in 2014” indicates neonates observed (= 1) or not observed (= 0). “ns” indicates we found no record of surveys conducted for neonates in past 3 years.
(XLSX)

S2 Table Table of data used in model selection analysis. (XLSX)
